# Metabolic–epigenetic crosstalk in latent autoimmune diabetes in adults: potential roles of lactate-induced histone lactylation in immune regulation and pancreatic β-cell fate

**DOI:** 10.3389/fendo.2026.1896333

**Published:** 2026-08-20

**Authors:** Wen Zhang, Xiaoran Wang, Bing He, Yongliang Xia, Zhijian Cao, Sumei Xu

**Affiliations:** 1 Department of Nephrology, The First Affiliated Hospital of Zhejiang Chinese Medical University (Zhejiang Provincial Hospital of Traditional Chinese Medicine), Hangzhou, China; 2 Department of Nephrology, The First People’s Hospital of Hangzhou Lin’an District, Hangzhou, China; 3 Department of Medical Administration, Development Center of Jiangshan City, Quzhou, China; 4 Department of Internal Medicine of Traditional Chinese Medicine,The First Affiliated Hospital of Zhejiang Chinese Medical University (Zhejiang Provincial Hospital of Traditional Chinese Medicine), Hangzhou, China; 5 Department of Radiology, The First Affiliated Hospital of Zhejiang Chinese Medical University (Zhejiang Provincial Hospital of Traditional Chinese Medicine), Hangzhou, China

**Keywords:** autoimmune diabetes, H3K18la, histone lactylation, LADA, metabolic–epigenetic crosstalk

## Abstract

Latent autoimmune diabetes in adults (LADA) is clinically between type 2 and type 1 diabetes with progressive autoimmune loss of β-cells and variable insulin dependence. Lactate was once thought to be a glucan byproduct and is now recognized as a signaling enzyme regulating gene expression via lysine lactylation. One of these marks is histone H3 lysine 18 lactylation (H3K18la), which is enriched at active enhancers and links immune genes. We examined literature and systematically distinguished direct LADA evidence from inferences from studies of type 1 diabetes, β-cell biology, autoimmune disease, and cancer. Present data posits elevated lactate and H3K9la in cell dedifferentiation, and H3K18la has stronger mechanistic support in immune regulation and enhancer activity. Cell-type-specific validation in longitudinal LADA cohorts and disease-specific experiments is lacking. We consider H3K18la a biologically plausible mechanism of LADA pathogenesis rather than an established driver. We emphasize that biomarker development or therapeutic translation will require strict LADA-specific evidence.

## Highlights

- Lactate functions as a metabolic–epigenetic signal through histone lactylation in LADA.
- Increased β-cell LDHA expression and elevated H3K9la connect glycolytic stress to β-cell dedifferentiation and loss of glucose-stimulated insulin secretion (GSIS).
- Immune-cell–derived lactate and H3K18la support a “trained immunity” hypothesis for LADA.
- Current evidence is indirect; cell-resolved validation is required before proposing these changes as biomarkers.

## 1 Introduction

**Figure 1:**
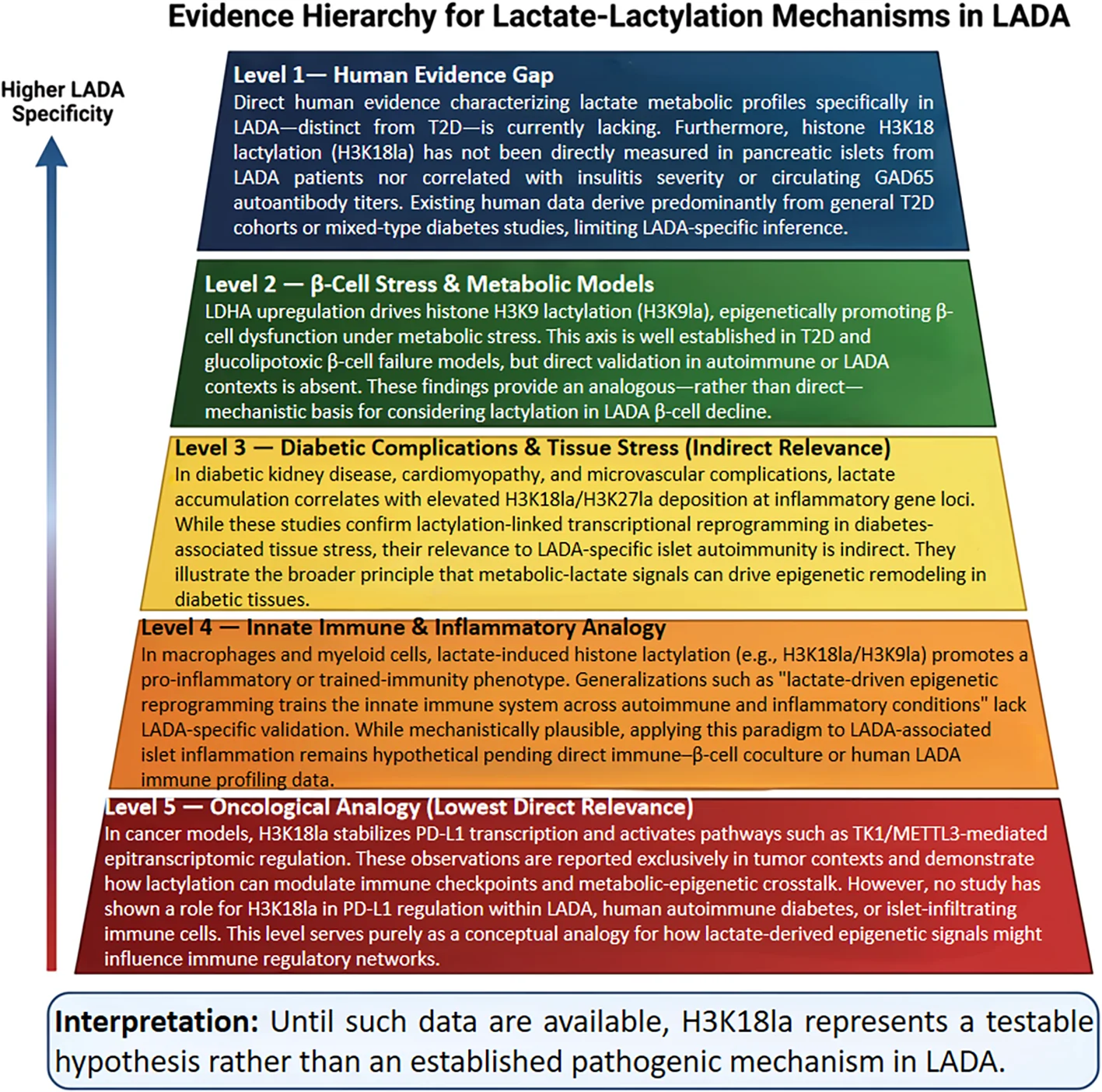
Evidence hierarchy for lactate-lactylation mechanisms in LADA. Vertical hierarchy: LADA specificity (top: highest, bottom: lowest). Colors indicate evidence types: blue (direct LAD Evidence: Green (direct diabetes/β-cell evidence: LDHA–H3K9la-mediated β-cell dedifferentiation and BCKA–LDHA suppression of glucose-stimulated insulin secretion); yellow (diabetic complication evidence: lactate/lactylation in kidney disease, corneal neuropathy, and microvascular injury); orange (autoimmune/inflammatory analogy: rheumatism, systemic lupus erythematosus, ulcerative colitis, and trained immunity); red (cancer mechanistic analogy: H3K18la feedback loops, immune escape, and glycolytic reprogramming). Left: higher (blue) to lower (red) LADA specificity. H3K18la remains a candidate mechanism requiring direct validation in LADA (see text). H3K18la, Histone H3 Lysine 18 lactylation; GSIS, Glucose-Stimulated Insulin Secretion; LADA, Latent Autoimmune Diabetes in Adults.

The term “latent autoimmune diabetes in adults” (LADA) refers to adult-onset islet autoimmunity (usually glutamic acid decarboxylase antibody) and insulin independence for at least six months after diagnosis (1). This definition, however, does not fully capture a disorder that follows a continuum from slowly progressive type 1 diabetes (T1D) to antibody-positive type 2 diabetes (T2D). Patients with high glutamic acid decarboxylase antibody (GADA) titers, multiple autoantibodies, and high-risk HLA genotypes follow a course similar to T1D, while patients with low titers, higher BMI, and insulin resistance follow T2D more closely. They differ in the extent of immune activation, metabolic stress, and residual β-cell function. Autoantibody status and HLA-associated genetic risk do not fully explain the heterogeneity of C-peptide loss or treatment response. Recent data suggest that metabolic reprogramming, altered glycolytic flux, and epigenetic remodeling play a role in the loss or preservation of β-cell identity and shape β-cell–immune cell crosstalk (2, 3). To assess the strength of evidence presented, we propose **Figure 1**, which separates direct clinical observations in LADA from indirect mechanistic evidence obtained from related models.

**Figure 2:**
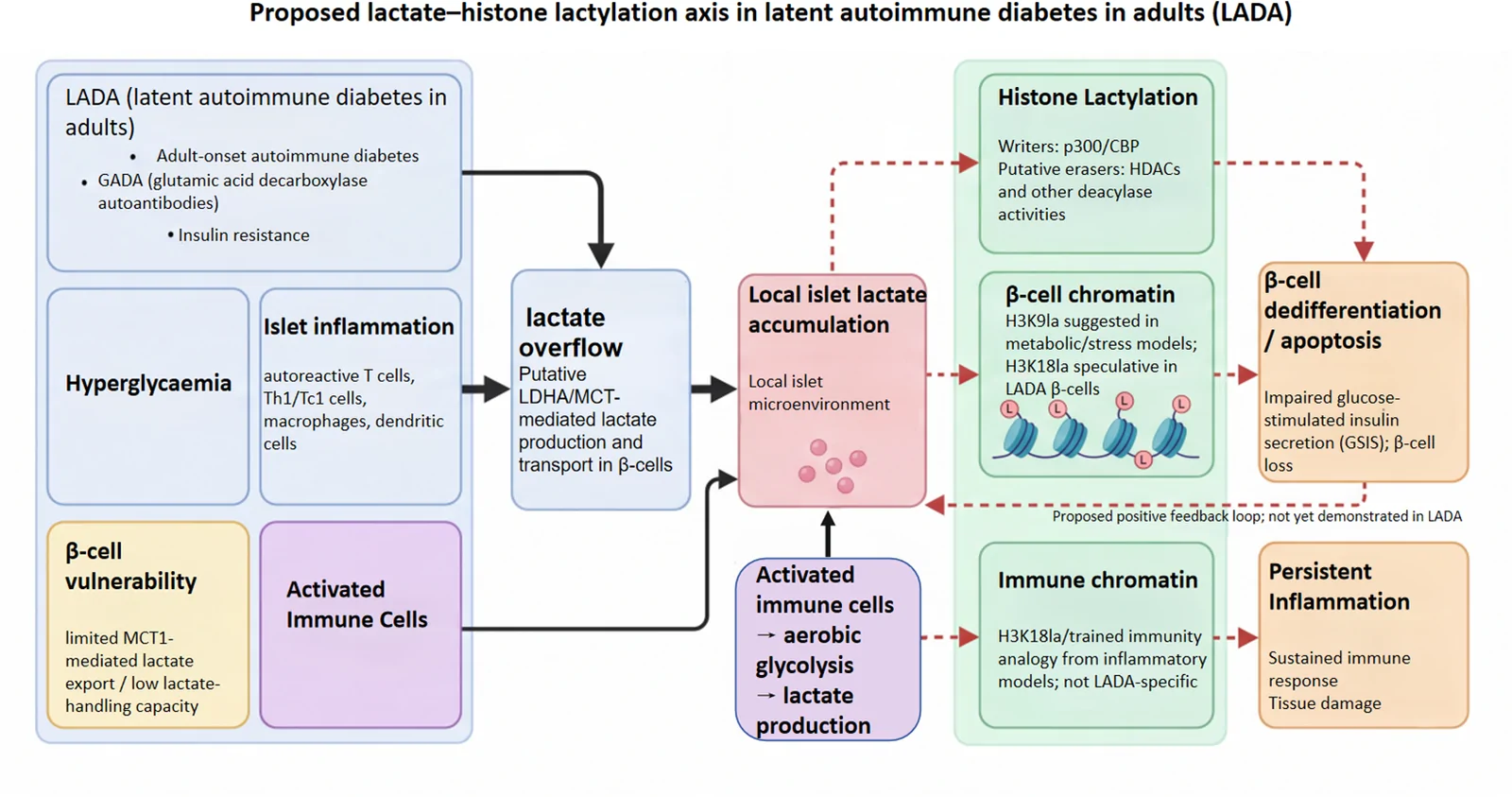
Lactate–histone lactylation axis for the onset of latent autoimmune diabetes in adults (LADA) and its possible role for immune dysregulation. Adult-onset autoimmune responses and environmental triggers such as viral infection disrupt local metabolic homeostasis. Cells turn to aerobic glycolysis and produce excess lactate accumulating in the pancreatic islet environment. High lactate can drive histone lactylation (H3K18la) and alter chromatin access and transcriptional programs. In pancreatic cells lactylation-associated epigenetic remodeling may promote dedifferentiation, inhibit GSIS, and increase apoptosis. In immune cells, lactylation may stabilize trained immunity and maintain proinflammatory transcriptional states. Persistence and lactate accumulation can induce a feed-forward circuit that increases metabolic stress and immune-mediated T-cell injury. This lactate–histone lactylation axis may provide a mechanistic link in LADA progression and suggest therapeutic targets for LADA and other autoimmune diseases like rheumatism. GSIS, Glucose-Stimulated Insulin Secretion; LDHA, Lactate Dehydrogenase A; H3K18la, Histone H3 lysine 18 lactylation.

In this context, H3K18la offers an easy experimental entry point for investigating metabolic-epigenetic regulation. Immune-cell activation is closely linked to metabolic rewiring, and metabolites like acetyl-CoA, succinate, and lactate can serve as substrates or modulators of chromatin-modifying enzymes, giving a plausible link between nutritional or metabolic states and long-term transcription programs (4–7). Three features of LADA make this framework appealing: first, the disease starts in adults with hyperglycemia or insulin resistance. Second, loss of β-cells is slower than classical T1D, suggesting years of chronic metabolic-immune interaction. Third, accumulation of lactate leads to H3K9la-dependent dedifferentiation of genes (2). We propose a lactate-histone lactylation axis linking metabolic stress to LADA pathogenesis (**Figure 2**) and integrating β-cell dedifferentiation and immune-trained immunity through H3K9la and H3K18la modifications.

Lactate-driven H3K18la has also been shown to induce STAT1- and TTK-dependent transcription feedback in cancer models (8, 9), indicating conceptual (though not disease-specific) evidence that lactate can transform transient metabolic stress into persistent change of the chromatin. Lactate is now known to be a metabolic fuel, redox buffer, and substrate for lysine lactylation (5, 10) and writes the post-translational modification (p300/CBP) and is erased by HDACs or sirtuins (7) and regulates macrophage polarization, T-cell adaptation, and trained immunity (11, 12). Lactate is also linked to β-cell dedifferentiation and impaired insulin secretion (2) and signals via GPR81/HCAR1 and glutathione-dependent redox (3). Whether systemic or islet-local lactate concentrations are higher in LADA and whether changes suffice to alter histone lactylation in cells or immune effectors is not known or measured directly in well-phenotyped cohorts.

## 2 LADA-specific clinical and biological background

### 2.1 Diagnostic criteria and limitations

LADA typically consists of three criteria: onset in adulthood (typically after age 30), being positive for at least one islet autoantibody, and insulin independence 6 months after diagnosis. Each of these criteria has limitations (13, 14). Adult-onset is not necessarily a standard indicator of a β-cell failure (a few people with type 1 diabetes suffer from rapid β-cell failure). Autoantibody positivity is not a standard indicator nor universally comparable. It depends on the test, population background, and the time period between disease onset and testing (15). The need for at least 6 months of an insulin-free period is also controversial, since it often reflects physicians’ treatment choices, access to healthcare, and local prescribing practices more than the patient’s true residual beta-cell function (16).

These limitations have direct implications for metabolic–epigenetic studies (17). A first-class diabetic patient, first labeled as type 2 and later GADA-positive, may have had long-term glucotoxicity and lipotoxicity before autoimmune diabetes was recognized (13, 15). ^A^ lean adult with high GADA titers and rapid C-peptide decline may have had a predominantly inflammatory course with minimal insulin resistance. Thus, studies on lactate metabolism or histone lactylation in LADA should divide the participants by autoantibody profile, C-peptide trajectory, BMI, insulin resistance, and HLA risk (1, 18).

### 2.2 Heterogeneity of LADA and implications for lactate biology

Heterogeneity in LADA supports the lactate–lactylation hypothesis. High-GADA or multi-autoantibody LADA indicates autoimmune activity and faster loss of β-cells (13, 19). Any lactate-dependent changes in epigenetics are likely to occur in activated immune cells, whether circulating or infiltrating islet cells. Low-GADA insulin-resistant LADA is exposed to higher metabolic stress: hyperglycemia, increased glycolytic flux, mitochondrial dysfunction, and dedifferentiation of β-cells (2). This may be particularly prone to LDHA activation and intracellular lactate accumulation in cells.

The same lactylation mark may also explain different biological meanings for different LADA subgroups. Higher H3K18la in monocytes may represent trained inflammatory memory, while higher lactylation in β-cells may represent metabolic exhaustion or dedifferentiation (16, 20). Cell-type–resolved signals from bulk peripheral blood mononuclear cells or serum will be hard to interpret.

### 2.3 Comparison of type 1 and type 2 diabetes

LADA combines the autoimmune characteristics of type 1 diabetes with the metabolic characteristics of type 2 diabetes and is in a heterogeneous intermediate position (13, 21). In T1D, β-cell destruction occurs due to strong autoimmunity (multiple autoantibodies), high-risk HLA genotype (HLA-DR3-DQ2 and HLA-DR4-DQ8), and prominent CD8+ T cell invasion (22). Direct evidence includes autoantibody positivity (GADA, IA-2A, ZnT8A), rapid C-peptide decline, and early insulin dependence. In T2D, insulin resistance, glucotoxicity, lipotoxicity, and chronic low-grade inflammation are major processes with preserved insulin or hyperinsulinemia in early stages (23). Rather, metabolic dysfunction is evidenced rather than autoimmunity.

The LADA spectrum is very broad. Patients with high GADA titers, multiple autoantibodies, and lean pathology are similar to T1D, and those with low titers, higher BMI, and insulin resistance are more closely related to T2D (13, 19). This provides different evidence levels: Level 1 (direct clinical evidence) for autoantibody and C-peptide pathology; Level 2 (indirect diabetes evidence) is derived from shared β-cell stress; Levels 3-4 (mechanistic inference) Lactate-lactylation pathways are extrapolated from T1D, T2D, or other autoimmune diseases (**Table 1**, **Figure 1**).

**Table 1:** Hierarchical evidence framework and interpretive guidelines for lactate-lactylation mechanisms across diabetes subtypes.

Evidence level	Description	Type 1 diabetes (T1D)	Type 2 diabetes (T2D)	Latent autoimmune diabetes in adults (LADA)	Interpretation guideline
Level 1: Direct Clinical Evidence	Quantitative measurements from disease-specific human cohorts	Islet autoantibodies (GAD65, IA-2), rapid C-peptide decline, HLA-DR3/DR4 susceptibility alleles	Insulin resistance, hyperinsulinemia, BMI >25 kg/m²	GAD65 autoantibody positivity, gradual C-peptide decline, adult-onset hyperglycemia	Use: ”Demonstrated in [disease] cohorts” (applies only to clinical phenotype; lactylation data absent across subtypes)
Level 2: Diabetes Model/Complication Validation	Mechanistic evidence from established diabetes models or complication tissues	Sparse β-cell lactylation data; lactate alterations noted in retinopathy/neuropathy contexts (24)	LDHA upregulation and H3K9 lactylation in pancreatic islets of db/db mice; elevated hepatic lactylation correlating with insulin resistance in HepG2 cells and diabetic hamsters (25)	No LADA-specific experimental data; existing β-cell lactylation insights extrapolated from T2D metabolic stress models (1, 24)	Use: ”Observed in diabetes models” (specify model type); avoid extrapolation to LADA without validation
Level 3: Diabetic Complication Analogy	Lactylation evidence from microvascular/macrovascular complication studies	Limited lactate metabolism reports in retinopathy/neuropathy (24)	H3K18la/H3K27la enrichment at Fas/HIPK2 promoters in diabetic kidney disease (DKD); lactylation-linked fibrosis in nephropathy and vascular calcification (1, 26, 27)	No LADA complication-specific lactylation data; relevance inferred solely from shared diabetic microvascular pathways (24)	Use: ”Suggested by diabetic complication studies” (specify tissue, modification site, and target gene)
Level 4: Autoimmune Disease Analogy	Mechanistic insights from non-diabetic autoimmune conditions	Partial overlap with RA/SLE pathways; H3K18la enrichment at TNF-α/IL-6 promoters in rheumatoid arthritis synovium (1)	Not applicable (non-autoimmune dominant pathophysiology)	Analogy to trained immunity and Th17-related lactylation in RA/ulcerative colitis; IFN-γ-rich milieu may engage NF-κB/STAT1 via lactylation (e.g., TRIM21 K345 lactylation stabilizing STAT1) (1)	Use: ”Proposed by analogy to autoimmune disease mechanisms” (emphasize lack of LADA validation)

Key distinctions: T1D: Strong Level 1 evidence for autoimmunity; limited lactylation data. T2D: Level 2 evidence for LDHA/H3K9la in islets; metabolic focus. LADA: Level 1 for clinical phenotype only; lactylation mechanisms at Level 4-5 (hypothesis stage).

Conclusion: H3K18la in LADA currently rests on Level 4-5 evidence. Advancing to Level 2 requires LADA-specific cell studies; advancing to Level 1 requires longitudinal cohort validation.

A lactate-histone lactylation framework is most relevant for LADA patients with chronic hyperglycemia, insulin resistance, and active autoimmunity, in which activated immune cells with aerobic glycolysis produce lactate, and metabolically stressed cells reactivate normally suppressed lactate production (12, 28, 29). Together, these processes could produce an islet microenvironment in which epigenetic remodeling might occur (although whether this happens in humans is unknown).

## 3 Histone lactylation biology

### 3.1 Structural basis of H3K18la

Histone H3 lactylation (H3K18la) is a lactylation where a lactyl group is covalently bound to lysine 18 of histone H3 (30, 31), changing local charge, affecting nucleosome contacts, and altering the recruitment of chromatin-binding proteins. Genome maps place H3K18la at CpG-rich active promoters and tissue-specific enhancers, where it often co-occupies with H3K27ac and H3K4me3 (32).

H3K18la has been detected in H19 in gastric cancer (33), NOD2 in bilirubin-induced astrocyte injury (34), TK1 in oral squamous cell carcinoma (35), and RhoA in sepsis-associated acute kidney injury (36). Early embryogenesis, H3K18la activates the zygotic genome activation function, which is not fully compensated by H3K27ac (37). Collectively, these results suggest that H3K18la is a functionally selective mark but does not play a pathogenic role in LADA.

### 3.2 Writers, erasers, and readers of lactylation

Histone acetylation is regulated dynamically. Acetyltransferase p300 is the writer of acetyl groups from acetyl-CoA to histone lysine residues (38). AARS1/2 have been proposed as noncanonical lactylation enzymes that may act independently of lactyl-CoA (7). Class I HDACs (especially HDAC1–3) and sirtuins (e.g., SIRT1–3) erase lactylation marks; e.g., SIRT1 downregulation in gastric cancer increases H3K18la accumulation and maintains the H19–glycolysis–H3K18la feedback loop (33, 38).

Reader mechanisms remain unclear. Brg1/SMARCA4 is a lactylation reader during pluripotent stem cell reprogramming and activates pluripotency and epithelial junction genes (39). TRIM33 is implicated as a downstream effector in lactylation-associated metabolic-epigenetic reprogramming in T-cell acute lymphoblastic leukemia (40). Is it possible that these writers, erasers, or readers function in human cells or immune cells relevant to LADA?

### 3.3 H3K18la versus H3K9la and other lactylation sites

One important distinction lies in H3K18la vs. other histone lactylation marks. The strongest β-cell evidence is H3K9la caused by LDHA activation: in diabetic islets, LDHA activation increases lactate and enriches H3K9la at dedifferentiation-associated sites such as Sox9, Hes1, and Aldh1a3, altering identity (2). This finding is relevant to diabetes but not specifically for H3K18la.

H3K18la plays an increasing role in immune regulation and enhancer function. In CD8+ T cells, H3K18la and H3K9la mark functionally relevant genes and share different genomic distributions (41). In triple-negative breast cancer, H3K18la, H3K9la, and H3K14la coaccumulate under glycolytic conditions and enhance chromatin accessibility together with H3K27ac (42). In pathological angiogenesis, the marks are controlled by a SEMA6A–p300–RHOA signaling axis (43). In trained immunity, H3K18la stays at distal regulatory regions for months after BCG stimulation (44). H3K19la and H3K9la are not interchangeable. In LADA, H3K9la offers stronger support for β-cell support, and H3K18la provides stronger evidence of immune function and enhancer activity. H3K18la could be considered a testable extension of existing evidence rather than a mechanism. **Table 2** lists histone lactylation sites for function, β-cell and immune evidence, and relevance to LADA: H3K9la and H3K18la are key marks.

**Table 2:** Histone lactylation sites relevant to diabetes and immunity.

Lactylation site	Main reported functions	β-cell/diabetes evidence	Immune evidence	Relevance to LADA
H3K9la	Active transcription; β-cell dedifferentiation genes	Strongest β-cell evidence via LDHA in diabetic models 2	Less defined	Indirect but important
H3K14la	Diabetic corneal nerve regeneration; tissue injury	Diabetic complication evidence 50	Limited	Exploratory
H3K18la	Active enhancers; trained immunity; glycolytic feedback loops	Weak/unknown in β cells	Stronger in trained immunity and immune/cancer models 16,28,41	Hypothesis core
H4K5la	Glycolysis-related feedback in HCC	Cancer/metabolic evidence 71	Limited	Conceptual
Non-histone lactylation	cGAS, PD-L1, Ezrin and other proteins	Disease-context dependent 20,42,43	Potential immune relevance	Requires profiling

## 4 β-cell Metabolic vulnerability to LADA

### 4.1 The β-cell lactate-disallowance behavior

Mature cells are metabolized by inhibition of disallowed genes LDHA and MCT1/SLC16A1. Low LDHA activity reduces pyruvate-to-lactate conversion, and low MCT1 expression limits inappropriate transport of lactate and pyruvate to the plasma membrane. Together, these constraints make insulin release reflect intracellular glucose metabolism rather than circulating lactate or pyruvate. Losing this disallowance behavior would blunt glucose sensing. Reactivation of LDHA removes pyruvate from mitochondrial oxidation into lactate and uncouples glycolysis from insulin exocytosis. Aberrant MCT1 expression may further disrupt stimulus–secretion coupling by allowing inappropriate lactate flux. Islet in LADA re-expresses LDHA, MCT1, MCT2, or MCT4. The question can be answered by studying donor islets classified by C-peptide trajectory.

### 4.2 Hyperglycemia-driven lactate accumulation

Chronic hyperglycemia increases glucose flux and can lead to lactate accumulation (45). Higher glucose levels stimulate glycolysis and lactate production (24). In hyperglycemia, especially local hypoxia, glycolysis can also be increased to produce lactate and histone lactylation (46). These observations do not directly show lactate accumulation in LADA islets but provide a mechanistic explanation for how chronic glucose excess might alter lactate metabolism and epigenetic regulation in β-cells. In LADA, progressive cell dysfunction combined with immune cells may create a local environment favorable for lactate enrichment. Addressing this hypothesis will require metabolomics studies of plasma, peripheral blood mononuclear cells, islet-derived extracellular vesicles, and, if available, human islet tissue.

### 4.3 LDHA-mediated glycolysis flux and β-cell dysfunction

In type 2 diabetes and animals, upregulated islet LDHA increases lactate accumulation, enriches H3K9la, and induces dedifferentiation markers such as Sox9, Hes1, and Aldh1a3, which decrease insulin secretion (2). Branched-chain α-keto acids activate LDHA directly and suppress GSIS, while deletion of specific β-cell LDHA restores secretory function under these conditions (47). However, LDHB-dependent lactate metabolism may support physiological fasting insulin release, suggesting that lactate handling in LADA β-cells may be adaptive rather than harmful (48). Whether LDHA is reactivated in LADA β-cells and whether this reactivation correlates with loss of PDX1/MAFA or faster C-peptide decline is still a question to answer.

### 4.4 Lactate shuttle between immune cells and islets

Activated T cells, monocytes, and macrophages depend on aerobic glycolysis and produce large amounts of lactate (49). In LADA, shifts in peripheral T cells (including expansion of Th1/Tc1-like effector populations) correlate with lower C-peptide (16) and can produce lactate locally in the islet. MCT4 is generally associated with lactate efflux from highly glycolytic immune cells, whereas MCT1 and MCT2 may mediate lactate uptake or exchange depending on the prevailing transmembrane concentration gradient. In addition, GPR81/HCAR1 offers a receptor-mediated route through which extracellular lactate can influence cellular signaling. Such a lactate shuttle may reshape the local metabolic milieu by modifying the NAD^+^/NADH balance, altering lactyl-CoA availability, and ultimately influencing histone lactylation in both immune cells and β cells. Notably, lactate-induced H3K18la has been implicated in the regulation of PD-L1 expression in tumour models (50). However, extrapolation of this mechanism to LADA requires caution. Unlike the tumour microenvironment, where attenuation of cytotoxic immunity may favor immune escape, the therapeutic aim in LADA is to re-establish immune tolerance while preserving functional β-cell mass. Reduced short-chain fatty acid production bacteria in the LADA gut microbiome implies broader systemic metabolic disturbance (51), whether these changes occur in lactate production or lactylation.

## 5 Epigenetic reprogramming mechanisms

**Figure 3:**
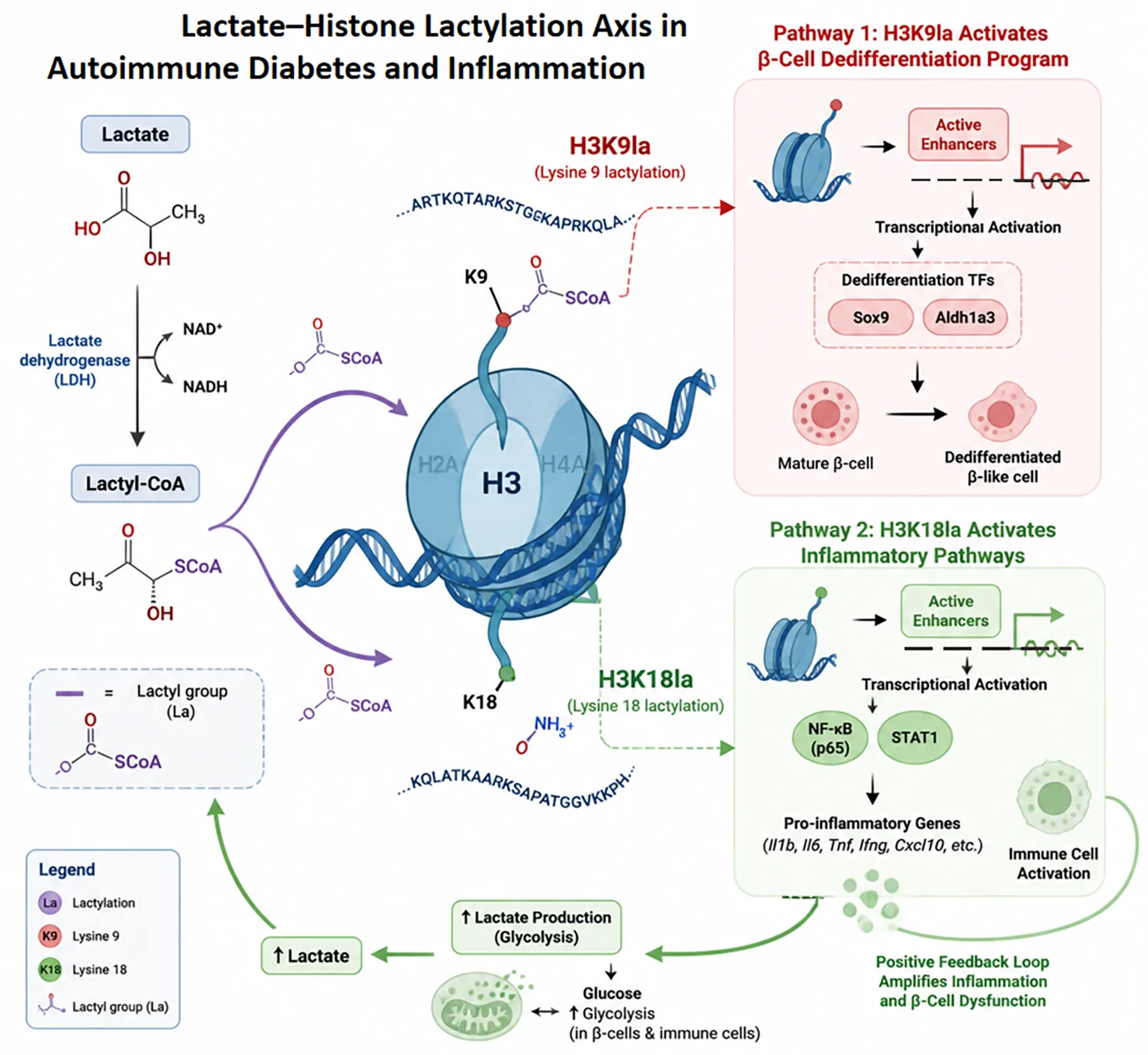
The lactate–histone lactylation axis in autoimmune diabetes. Metabolic reprogramming raises intracellular and extracellular lactate. Lactate is converted into lactate by acetyl-CoA synthetase (ACS) or equivalent processes. Lactyl-CoA is used for lactylation (a post-translational modification targeting mostly the lysine residues of histone H3). The panel summarizes two key regulatory pathways driven by site-specific lactylation. Pathway 1: H3K9la opens the chromatin and activates transcription factors (Sox9 and ALDH1A3), leading mature beta cells toward a dedifferentiated beta-like state and hindering glucose-stimulated insulin secretion (GSIS). Pathway 2: Histone H3K18la activates enhancers and upregulates proinflammatory genes IL1β, TNF-α, and IFNG by NF-κB (p65) and STAT1. Activated immune cells and dysfunctional β-cells increase glycolysis and lactate production, creating a positive feedback loop that keeps histone lactylation, inflammation, and loss of β-cells. GSIS, glucose-stimulated insulin secretion; H3K9la, histone H3 lysine 9 lactylation; H3K18la, histone H3 lysine 18 lactylation; LDHA, lactate dehydrogenase A; MCT, monocarboxylate transporter.

**Figure 3** shows that lactate is converted to lactyl-CoA and that lactyl-CoA modifies histone H3 at K9 and K18. H3K9la promotes β-cell dedifferentiation (Sox9 and ALDH1A3); H3K18la induces inflammation (NF-κB/STAT1). Together, these changes lead to a lactate–glycolysis positive feedback loop supporting a central LADA hypothesis, which needs ChIP-seq or CUT&Tag validation.

### 5.1 H3K18la-mediated chromatin remodeling in cells: hypothesis

H3K18la is enriched at active promoters and at tissue-specific enhancers in multiple cell types (32); high glucose exposure can change histone modifications and alter transcription (45); increased lactate production in pancreatic cancer-initiating cells raises H3K18la levels and sustains stemness-associated epigenetic reprogramming (52). Together, these results suggest H3K18la may be mediating metabolic stress-driven fate plasticity (52).

Direct evidence is limited in β-cells; the strongest β-cell data concern H3K9la downstream of activation of LDHA (2). Any model using H3K18la in LADA dedifferentiation must be explicitly posed as a hypothesis. One possibility is that chronic hyperglycemia and inflammation drive LDHA-dependent lactate accumulation, which raises histone lactylation at regulatory regions. In such a scenario, H3K9la could activate loci that are dedifferentiated, and H3K18la could modulate active enhancers related to stress response, antigen presentation, or β-cell identity genes such as PDX1, MAFA, INS, SLC2A2, and GCK. ChIP-seq, CUT&Tag, or CUT&RUN will be used to test this hypothesis.

### 5.2 Lactylation-dependent networks

H3K18la may modify transcription by recruiting reader proteins and co-activating marks. In T-cell acute lymphoblastic leukemia, H3K18la and H3K27ac promote metabolic-epigenetic reprogramming with TRIM33 acting downstream (40). In colorectal cancer liver metastasis, H3K18la is abundantly expressed at promoters of pro-metastasis genes in a TROP2–HIF-1α transcriptional axis (53). Cancer-derived examples provide mechanistic analogs rather than direct evidence for LADA.

The important empirical question is whether lactylation-dependent networks function in cells or immune β-cells of LADA patients. Candidate pathways in cells include dedifferentiation, endoplasmic reticulum stress, interferon signaling, antigen presentation, chemokine production, and increased apoptotic susceptibility. In immune cells, H3K18la could regulate cytokine loci, memory enhancers, or metabolic adaptation programs. Such networks should be defined directly experimentally rather than by analogy.

### 5.3 Positive feedback between lactate metabolism and histone acetylation

Positive feedback between glycolysis and chromatin remodeling is a common theme in lactylation biology. In adipose stem cells, H3K18la activates HK2, which enhances glycolysis and lactate production (54); in pancreatic ductal adenocarcinoma, H3K18la activates TTK and BUB1B expression (9), and TTK phosphorylates LDHA to increase lactate production (7); and in gastric cancer, loss of SIRT1 induces an H19–glycolysis–H3K18la feedforward loop (34).

Similar architecture may also be implemented for LADA, though this has not been proven yet. Chronic hyperglycemia and immune inflammation could increase LDHA activity in cells and induce lactate accumulation, which increases histone lactylation. Increased H3K9la and H3K18la could activate glycolytic genes, inflammation pathways, and stress response pathways, and thus stimulate lactate production and immune activation. Such a feed-forward loop could explain the characteristic slow but progressive failure of LADA. Importantly, we obtain testable predictions: measured correlation between lactate concentration, LDHA activity, H3K9la and H3K18la activity, inflammation gene expression, and C-peptide decline.

## 6 Lactylation in immune regulation

### 6.1 Innate immune memory and trained immunity

“Training immunity” refers to the permanent functional reprogramming of the immune cells after a stimulus. BCG vaccination raises H3K18la levels of monocytes and macrophages, localizing to the region where it is readily available to the chromatin and can last up to 90 days (44). Pharmacological inhibition of lactate production or blocking histone lactylation can limit trained immunity, and variation in genetic LDHA and EP300 can influence the differences between individuals (44). Independently, lactate has been shown to be a metabolic substrate for trained immunity as it fuels the tricarboxylic acid cycle (11).

Is trained immunity linked to LADA disease? Repetitive metabolic stress (hyperglycemia, free fatty acids) or microbial stimulus could stimulate monocytes and macrophages and induce stable H3K18la marks at inflammatory enhancers. Whether such programming uses β-cell antigens, persists long-term in islet-infiltrating macrophages, or correlates to C-peptide decline is not clear. Maladaptive training programs may perpetuate low-grade islet inflammation, or some programs may promote tissue repair. Direct analysis of monocyte and macrophage lactylation states in LADA may be warranted.

### 6.2 T-cell function and immune tolerance

Lactylation also affects adaptive immunity. In CD8+ T cells, histone acetylation determines genes responsible for metabolism and effector function (41). The tumor microenvironment shows that lactate accumulation remodels myeloid and T-cell states, strengthens immune checkpoint barriers, and establishes self-sustaining metabolic feedback loops (55). Lactate-related modifications can regulate immune sensors like cGAS and balance inflammation and tolerance (56). PD-L1 lactylation status also influences tumor growth and anti-tumor immune response (57).

Our results show that lactate and lysine lactylation modulate T cell biology but should not be interpreted as cancer-style immune checkpoint blockade strategies for LADA since inhibition of PD-1/PD-L1 can trigger autoimmune diabetes and β-cell destruction. In LADA, an ideal immunomodulatory strategy would attenuate autoreactive Th1, Th17, and Tc1 responses while preserving Treg stability and avoiding clinically relevant impairment of host defense. Given the rapid recall capacity of memory T cells, as well as the contribution of trained innate immune programs to amplified inflammatory responses (58), lactylation may plausibly participate in directing autoreactivity in LADA. However, this possibility is still speculative, as direct evidence in LADA is currently lacking.

### 6.3 Macrophage polarization: insights plus caveats from cancer studies

Histone lactylation has been progressively acknowledged as a mechanism capable of influencing macrophage plasticity. In tumor settings, lactate-driven histone lactylation has been reported to promote M2-like transcriptional programs, dampen antitumor immune activity, and facilitate immune escape (59, 60). These observations offer a useful conceptual framework, but their relevance to autoimmune diabetes should be interpreted with caution. Unresolved Biological Paradox (Cancer vs. Autoimmunity): The manuscript heavily relies on cancer models to explain H3K18la mechanisms. However, in tumors, lactylation drives immunosuppression and immune evasion, whereas in LADA, you hypothesize it drives hyper-inflammation and autoimmune destruction. The text fails to adequately explain how the exact same metabolic mark can produce diametrically opposite immunological outcomes under similar lactate contexts. The immunology of LADA is different from that of cancer. In cancer, lactylation promotes an immunosuppressive tumor microenvironment, e.g., H3K18la-dependent M2 macrophage polarization, PD-L1 upregulation, and CD8+ T cell dysfunction. In LADA, however, the pathophysiology must suppress autoreactive responses while maintaining protective host defense.

Lactylation exhibits a striking microenvironment-dependent functional plasticity. In hypoxic, TGF-β-rich tumor niches, lactate accumulation and H3K18la induce immunosuppression by upregulating ACAT2 and skewing macrophages toward M2. In contrast, in the LADA islet microenvironment, characterized by chronic hyperglycemia, IFN-γ from autoreactive Th1/Tc1 cells, and β-cell danger signals, the same H3K18la modification targets NF-κB and STAT1 to increase proinflammatory gene expression rather than suppress immunity (61). Single-cell analyses further show that peripheral immune cells in LADA maintain a controlled “inflammation–inhibition balance” with low NF-κB/EGFR signaling but healthy JAK/STAT activity and HLA-C–KIR regulatory checkpoints (21).

This context duality resolves the apparent paradox: lactylation is not intrinsically pro- or anti-inflammatory. Its functional outcome is determined by local factors, such as cytokine composition (IFN-γ vs. TGF-β), oxygen tension, lactate concentration, cell type specificity, and disease stage. This context dependence is therefore important for designing medical strategies that target lactylation in autoimmune diabetes and cancer.

Evidence from other metabolite-sensitive epigenetic pathways further supports the wider principle that immune-cell fate can be determined by metabolic cues. For example, short-chain fatty acids can modulate macrophage polarization owing to inhibition of histone deacetylases, showing how metabolites may influence immune function through epigenetic regulation (62). In LADA, macrophage lactylation is likely context-dependent. Depending on the concentration of lactate, the cytokine environment, or disease stage, lactylation may increase the production of inflammatory cytokines, antigen presentation, tissue repair, or immune suppression. BCG-trained macrophages retain H3K18la associated with the activation of the inflammatory gene (44), and tumor-associated macrophages (TAMs) of pancreatic cancer use glycolysis, PCAFH3K18laCXCL1, to drive neutrophil invasion and an immunosuppressive environment (63, 64). Recent evidence also shows that PCAF acts as a histone lactyltransferase in pancreatic cancer and activates CXCL1 transcription directly via H3K18 lactylation (64). Conversely, in inflammatory and autoimmune conditions, histone lactylation can lead macrophages to an M2-like reparative function (see exercise-induced MeCP2 K271 lactylation inhibits atherosclerosis) (65). These results suggest that lactylation in LADA is not necessarily pro- or antiinflammatory but rather may oscillate between protective and pathogenic roles depending on local metabolic immune signals. Therefore, macrophage lactylation in LADA should not be considered to be detrimental or beneficial; cell-type-resolved and stage-specific studies are needed.

## 7 Diagnostic and therapeutic implications

### 7.1 H3K18la as a candidate exploratory biomarker

H3K18la is associated with disease progression or treatment resistance in hepatocellular carcinoma, gastric cancer, pancreatic ductal cancer, and papillary thyroid carcinoma (66). Lactate accumulation and histone lactylation (including H3K14la) correlate with reduced tissue function (67). In sepsis immunosuppression, H3K18la regulates autophagy gene expression (68). Collectively, these results support the hypothesis that lactylation acts as a metabolic-epigenetic marker of stress in cancer and acute inflammation, but H3K18la is not a validated biomarker of LADA. There is no direct evidence that H3K18la levels measured in peripheral blood mononuclear cells, serum extracellular vesicles, or pancreatic islets differentiate LADA from type 1 diabetes, type 2 diabetes, or healthy controls. No studies have associated H3K18la with autoantibody titers, C-peptide reserve, HbA1c trajectories, or time to insulin dependence in LADA cohorts. The evidence hierarchy (**Figure 1**) places H3K18la at Level 3-4 (mechanistic inference from related diseases), not Level 1 (direct LADA clinical evidence). Validation as a LADA biomarker would require the following: Cross-sectional measurements between diabetic subtypes and healthy controls Cross-sectional measurements across diabetic subtypes and healthy controls.

### 7.2 Targeting LDHA and lactate metabolism

LDHA occupies a central position at the interface between glycolytic flux, lactate generation, and lactate-dependent epigenetic regulation. Evidence from diabetic complications suggests that excessive LDHA activity may have tissue-specific pathogenic consequences. In diabetic neuropathy, LDHA-driven lactylation has been shown to impair corneal nerve regeneration, whereas LDHA knockdown reactivates regenerative signaling pathways (67). Similarly, in diabetic kidney disease, LDHA-associated lactate accumulation and tissue acidosis contribute to renal fibrosis and mitochondrial dysfunction. In pancreatic β cells, induction of LDHA by branched-chain keto acids suppresses glucose-stimulated insulin secretion, while genetic deletion of LDHA restores secretory capacity (47). Collectively, these data support the view that LDHA is not simply a metabolic enzyme but may also function as a regulator of cellular phenotype through lactate-linked metabolic and epigenetic mechanisms. In cancer and kidney fibrosis models, LDHA inhibition or lactate-targeting nanomedicine reduces pathological lactate levels (33, 69). Systemic LDHA inhibition risks because LDHA is essential for muscles, erythrocytes, immune defense, hypoxic tissue adaptation, and hepatic lactate cycling. In LADA, LDHA-targeted strategies should be used as research tools rather than as clinical treatments. Clinical translation will require cell-protective delivery systems, immune-cell–specific modulation, or stage-limited interventions instead of chronic systemic blockade.

### 7.3 Epigenetic modulators and immune-tolerance strategies

Lactylation is reversible by writers and erasers such as p300/CBP, HDACs, and sirtuins (1, 38, 40). In cancer models, inhibition of LDHA, p300, or glycolysis reduces H3K18la and improves checkpoint immunotherapy (42, 70). In rheumatism models, inhibition of Th17 differentiation via LDHA/H3K9la/NFATC2– (71) and inhibition of CD8+ T cell activation by targeting LDHA in transplantation models (72). The therapeutic rationale in LADA is different from that in oncology because it is to restore immune tolerance and preserve β-cells rather than to increase cytotoxicity. Thus, optimal strategies are likely to combine antigen-specific tolerance induction with low-dose IL-2, anti-CD3 therapy, CTLA4-Ig, or cytokine modulation instead of PD-1/PD-L1 blockade. Metabolic–epigenetic modulation may complement these interventions by dampening pathogenic activation and maladaptive trained immunity, but this idea needs validation in human cells and experimental models. **Table 3** lists therapeutic targets on the lactate-lactylation axis, including mechanisms, benefits, risks, and LADA evidence levels.

**Table 3:** Potential therapeutic targets in the lactate–lactylation axis.

Target	Mechanism	Potential benefit	Major risk	LADA evidence level
LDHA	Reduces lactate production	May preserve β-cell metabolic identity; reduce lactylation	Systemic metabolic toxicity; impaired muscle/immune adaptation	Indirect
MCT1/MCT4	Alters lactate transport	May disrupt pathological lactate shuttle	May impair normal tissue lactate exchange	Speculative
GPR81/HCAR1	Modulates lactate signalling	Could tune immune or β-cell responses	Context-dependent effects	Speculative
p300/CBP	Reduces lactylation writing	May suppress abnormal transcriptional activation	Broad effects on acetylation and transcription	Speculative
HDAC1–3	Delactylation and deacetylation	Potential epigenetic resetting	Highly non-specific; immune and β-cell toxicity	Speculative
SIRT1/3	Delactylation and metabolic regulation	May restore metabolic–epigenetic balance	Pleiotropic effects	Indirect
Tolerogenic immunotherapy	Anti-CD3, CTLA4-Ig, low-dose IL-2	Restores immune tolerance	Timing and patient selection critical	Conceptually relevant
Combined metabolic–immune strategy	Stage-specific modulation	May protect β cells and reduce autoreactivity	Requires careful safety testing	Future direction

## 8 Controversies and unresolved questions

### 8.1 Tissue specificity of lactylation effects

Lactylation effects are context-dependent and differ in different cell types and tissues. For example, in cancer, immune cells, and autoimmune disorders, lactylation modulates inflammation, remodels metabolism, and influences cell-fate decisions (29). In pancreatic ductal carcinoma, H3K18 lactylation changes the transcriptional activity of TTK and BUB1B 9, and in macrophages, lactylation targets ACAT2 and promotes M2-like polarization (63). These effects cannot be directly applied to pancreatic islets or to LADA. β-cells depend heavily on mitochondrial oxidative metabolism, have limited glycolytic flexibility, and exhibit relatively low basal LDHA and MCT1 (73). These constraints change how β-cells handle lactate and thus alter the interpretation of lactylation marks. In other words, the same lactylation modification can produce different transcriptional and functional results for β-cells, monocytes, macrophages, and T cells. Hence, cell–type–specific functions of lactylation are important before attribution of mechanistic relevance to LADA. Future studies should distinguish lactylation events in immune compartments from those in cells or islet microenvironments because biological implications may differ markedly depending on whether the immune compartment or islet function is a cell–type–specific function of lactylation events in immune compartments with different biological effects.

### 8.2 Divergent roles of lactylation in autoimmunity and cancer

Lactate accumulation and protein lactylation exert significantly different immunological effects in cancer and in autoimmune disease. In tumors, a lactate-rich microenvironment and higher lactylation drive immunosuppressive macrophage remodeling, suppress T cell effector functions, and help immune evasion (55, 74, 75). In autoimmune disease, increased lactate stimulates proinflammatory cytokine production, stimulates immune cells to enter, and causes tissue damage (29, 49). This paradox can be solved if lactylation is seen as a context-sensitive regulatory modification rather than as a categorical pro- or antiinflammatory signal. Multiple microenvironmental factors determine the function of lactylation:

1). Oxygen availability: Tumor hypoxia stabilizes HIF-1α, which cooperates with lactylation to activate immunosuppressive programs (PD-L1, VEGF). Normal or intermittently hypoxic islets may not sustain HIF-α1 stabilization, thereby allowing lactylation to activate other transcription factors. 2). Cytokine environment: TGF-β and IL-10 in the tumor synergize with lactylation to suppress effector immunity. In LADA, IFN-γ, TNF-β, and IL-1 from autoreactive T cells create a pro-inflammatory niche in which lactylation may increase rather than attenuate immune activation via the NF-κB and STAT1 pathways (44). 3). Cell composition and metabolic state: Tumor-associated macrophages rely on lactate as an energy source and tolerate high lactate via MCT1 upregulation. In contrast, islet-resident macrophages may respond to lactate as a damage-associated metabolic signal, triggering inflammasome activation. 4). Lactate concentration and exposure time: Chronic millimolar-range lactate in solid tumors reprograms chromatin, while lactate spikes in LADA postprandial hyperglycemia may produce episodic rather than stable epigenetic marks. Lactylation is relevant in adult-onset autoimmune diabetes such as LADA because metabolic stress and autoimmune activity are interrelated.

These effects are unlikely to result from lactylation alone. In fact, they are likely to be related to several other factors: lactate concentration, exposure duration, oxygen availability, the cytokine environment, cell composition, and metabolic state. Thus, lactylation should be considered as a context-sensitive regulatory modification rather than categorically pro- or anti-inflammatory. In adult-onset autoimmune diabetes, such as LADA, lactylation may be relevant because metabolic stress may be linked to autoimmune activity. Multiple nonmutually exclusive mechanisms could explain this link. Lactylation may reinforce inflammatory memory in immune cells, alter auto-active T cells, or increase β-cell stress response during metabolic overload (76). Recent work showing stage-dependent shifts in islet macrophage metabolism during the development of autoimmune diabetes (64) and lactate-mediated regulation of immune checkpoints in tissue-resident lymphocytes (65) supports this context-dependent model. Whether these effects are pathogenic, compensatory, or dependent upon disease stage is unknown. The answer will depend on disease stage-specific cell type-resolved studies on lactylation in immune cells and pancreatic islets.

### 8.3 Technical challenges in lactylome profiling

Accurate profiling of protein lactylation remains challenging. Lactylation occurs infrequently, and it is often difficult to distinguish it from structurally related lysine acylations. Antibodies against site-specific lactylation marks, such as anti-H3K18la, must be validated before use in chromatin or immunodetection studies. Cell sources of lactylation substrates and biosynthesis of lactyl-CoA are also incompletely defined (1). Another limitation is the composition of current lactylome data. Most papers used tumor cells, cancer tissues, or animal models. Data from human pancreatic islets, especially LADA, are scarce (9, 63). Human samples are difficult to obtain and are heterogeneous and often small in quantity, which limits high-resolution profiling. Lactylation is likely dynamic and responsive to metabolic and inflammatory signals. Transient changes in glucose fluctuations, hypoxia, or immune activation can be missed in bulk analyses.

Current technologies have limitations. Mass spectrometry has high specificity but does not provide routine single-cell or time-resolved lactylome resolution. Chromatin-based techniques such as ChIP-based methods, CUT&Tag, and related ones require large input material and depend heavily on antibody quality. These limitations explain why the lactylation mechanism in LADA is only inferred rather than directly demonstrated (76). Future progress will require standard antibody validation, isotope tracing to map lactate-derived carbon flow, targeted mass spectrometry for site-specific quantification, and low-input chromatin profiling methods such as CUT&Tag. This will be crucial for integrating these tools with single-cell multiomics tools to resolve lactylation dynamics between β-cells, immune cells, and other components of the islet microenvironment.

## 9 Research roadmap and future perspectives

### 9.1 Single-cell multiomics

Single-cell multiomics provides the resolution required to unravel lactate-lactylation mechanisms in LADA. scRNA-seq, ATAC-seq, and CUT&Tag have shown that lactate-driven H3K18la remodels chromatin accessibility and transcriptional programs in cancer models (35). Similar results could be obtained in sorted PBMC subsets, iPSC-derived β-like cells, pseudo-islets, or donor islets from LADA patients. The aim is to map H3K18la and H3K9la at β-cell identity genes, antigen-presentation loci, interferon-response elements, and inflammatory enhancers; to link lactylation patterns to LDHA and MCT expression; and to identify immune subsets with trained or exhausted genes (77). Single-cell metabolic profiling coupled with lactyl-proteomics may help to resolve lactate shuttling between immune cells and β-cells. Since H3K18la preferentially marks active promoters and enhancers (32), cell-type-specific distribution may also help define regulatory circuits relevant to LADA.

### 9.2 LADA-relevant experimental models

One challenge is the lack of animal models that reproduce adult-onset, slowly progressive autoimmune diabetes and heterogeneous LADA. Future models could combine autoimmune-susceptible backgrounds (NOD, HLA-DQ8) with conditional manipulations of lactylation regulators such as p300/CBP, HDAC1-3, SIRT1/3, or LDHA (1). β-cell–specific reactivation of LDHA or MCT1 would test whether disruption of the lactate-disallowance phenotype is involved with autoimmune failure (78). Similar β-cell-specific or immune-cell-specific approaches might test whether lactylation drives or simply accompanies autoimmune β-cell loss (79). Pharmacological tools such as dichloroacetate might be useful experimentally but need cautious interpretation (79).

### 9.3 Clinical translation of metabolic–epigenetic interventions

Translating lactate–lactylation biology into LADA therapies requires clarifying disease stage, patient subgroup, tissue specificity, time of intervention, and safety. LDHA inhibition, p300 modulation, and sirtuin activation might reduce maladaptive lactylation (9), but these targets are fundamentally essential, and nonselective modulation could disrupt adaptive lactylation, protective immunity, or system metabolism. An example of practical translation would be biomarker-guided patient selection; cell-type-specific delivery and immunotolerance-promoting therapies and short-term intervention in early autoimmune activation or rapid C-peptide decline would be preferred over prolonged suppression. Lactylation-based prognosis frameworks for ovarian cancer and hepatocellular carcinoma show that multi-omics can reveal actionable targets (80). Similar frameworks for LADA could include H3K18la and H3K9la profiles, lactate metabolism index, autoantibodies, C-peptide, HLA genotype, BMI, and immune phenotype.

## 10 Concluding remarks

### 10.1 Key mechanistic insights

Lactate is a signaling molecule that can remodel chromatin by histone lactylation. Diabetes-relevant β-cell models of the disease induce lactate production and H3K9la to dedifferentiate insulin and lower insulin secretion; immune and cancer models regulate active enhancers, trained immunity, and glycolysis-linked transcriptional feedback. These results support a plausible, though unproven, model in which lactate-dependent histone lactylation is involved in LADA development. The strongest evidence for LADA is clinical and immunological: adult-onset autoimmunity, variable C-peptide decline, altered T-cell subsets, and metabolic heterogeneity (Level 1 direct evidence). There is currently a lack of direct evidence demonstrating lactate accumulation, LDHA/MCT dysregulation, or H3K18la elevation specifically in LADA patients or islets; therefore, the lactate–H3K18la axis should be regarded as a testable hypothesis (Level 3–4 mechanistic inference) rather than an established pathogenic mechanism. **Table 1** summarizes the evidence hierarchy for lactate-driven lactylations in LADA and provides strength of interpretation and suggested wording for each level (25–27).

### 10.2 Therapeutic roadmap for precision medicine

A precision medicine strategy for LADA should incorporate metabolic stress, immune dysregulation, and β-cell vulnerability. LDHA, lactate transporters, GPR81, p300/CBP, HDACs, and sirtuins may be targets for targeted study. Natural or pharmacological agents that lower lactate production or reduce H3K18la have been used in cancer models, but their applications to LADA are unknown. Future therapeutic development should focus on three priorities: preserve β-cell identity and glucose responsiveness; restore immune tolerance rather than cytotoxicity; and minimize systemic metabolic toxicity. Single-cell multiomics, human β-cell platforms, LADA-stratified cohorts, and genetically engineered models will need to determine whether targeting lactate-induced lactate can safely delay β-cell failure; if validated, this framework could provide a new approach to combine metabolic modulation, epigenetic resetting, and immune tolerance in LADA.
